# Step-by-Step Cardioneuroablation Approach in Two Patients with Functional Atrioventricular Block

**DOI:** 10.4274/balkanmedj.galenos.2019.2019.9.47

**Published:** 2019-10-28

**Authors:** Tolga Aksu, Tümer Erdem Güler, Kıvanç Yalın

**Affiliations:** 1 Clinic of Cardiology, University of Health Sciences, Derince Training and Research Hospital, Kocaeli, Turkey; 2 Department of Cardiology, Uşak University School of Medicine, Uşak, Turkey

**Keywords:** Ablation, adult, cardiovascular disease, ganglionated plexus, parasympathetic, syncope

## Abstract

Parasympathetic overactivity may cause functional atrioventricular block episodes and necessitate pacemaker implantation in symptomatic cases and those refractory to conventional therapies. In these patients, if it can be clearly demonstrated that there is no structural damage in the conduction system, elimination of the vagal activity based on radiofrequency catheter ablation of main ganglionated plexi around the heart, which is called as cardioneuroablation, might be a rational approach. In this review article, we try to discuss patient selection and procedural steps suitable for cardioneuroablation based on two patients with functional atrioventricular block.

As a rare form of atrioventricular block (AVB), vagally mediated or functional AVB, is characterized by a sudden change from apparently physiological atrioventricular (AV) conduction to transient second- or third-degree heart block, due to parasympathetic influence on cardiac conduction (1). Paroxysmal AVB episodes may lead to poor quality of life, frequent syncopal episodes, and syncope without prodromal symptoms posing the patient to a risk of trauma (2,3). In those cases, elimination of the vagal innervation by radiofrequency catheter ablation of the main ganglionated plexi (GPs) might be a rational approach, because GPs demonstrate a tendency to settle more in certain regions and contain neuronal bodies of parasympathetic system. Usage of the technique in bradyarrhythmia was attempted by Pachon et al. (4) for the first time and termed it as cardioneuroablation (CNA). However, there is still no consensus on the most adequate technique and selection of patients with AVB acceptable for CNA (4,5,6,7). Present article is dedicated to discuss suitable patient selection and procedural steps for CNA based on two complicated patients with functional AVB.

## CASE 1 PRESENTATION

**Figure 1:**
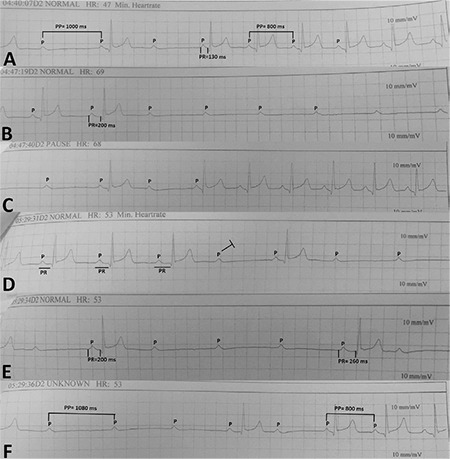
**a-f. Holter ECG tracings of case 1.** A clear sinus rate slowing during AVB episode and a PR interval prolongation before AVB are seen. Both findings are compatible with vagally-mediated AVB. Also, high-degree AVB is seen in B, E, and F tracings. For details, please see the text. AVB: atrioventricular block

**Figure 2:**
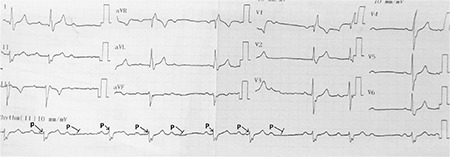
**Rest ECG of case 1.** Mobitz Type I AVB seen on rest ECG. AVB: atrioventricular block; ECG: electrocardiography

A 61-year-old male was referred to our clinic for implantation of a permanent pacemaker because of symptomatic AVB episodes. Within the last 3 months, the patient had experienced recurrent episodes of dizziness and one syncopal episode. Repeated Holter recordings demonstrated variable degree AVB (Figure 1). Mobitz type I AVB was detected on his admission electrocardiogram (Figure 2).

### Determining whether the block is functional

To demonstrate functional nature of AVB, electrocardiogram characteristics before, during, and after the episode should be carefully examined on continuous Holter tracings. In vagally mediated type, AVB is usually preceded by sinus node slowing, and the sinus rhythm during AVB is slow and instable (8). A progressive PR interval prolongation just before AVB may be noticeable. Wenckebach type, 2:1 second-degree, or high-degree AVBs can be recognized during the episode. However, in other types of paroxysmal AVBs, the PR interval remains unchanged and sinus rate increases or remains unchanged (9).

### Case 1 presentation (continued)

In the present case, there was no rise in the sinus rate during AVB episodes. On the contrary, a clear sinus rate slowing was seen during the episode (Figure 1). Moreover, PR interval prolongation before AVB could be easily recognized on comparison of the intervals (Figure 1a and 1b). All these findings were in favor of functional nature of AVB.

### Suitability of the case for cardioneuroablation

There is no well-studied treatment option for patients with functional AVB. Despite lack of enough evidence and limited efficacy, cardiac pacing is suggested by European guideline in patients ≥40 years with significant complaints and documented symptomatic pauses (10).

Theoretically, CNA mimics sinoatrial and AV nodal effects of atropine (4,5,11,12). Therefore, to define procedural endpoint and to predict potential results of ablation, pre-procedure atropine response should be checked in all cases at least 24 hours prior. However, there is no consensus on atropine dosing between the studies (4,5). In our previous study protocol, atropine bolus injection with 0.04 mg/kg was used (5). According to the latest guideline, atropine should be used with 1 mg initial dose and repeated until desired heart rate is obtained in every 3 to 5 min, with a maximum dosage of up to 3 mg in case of hemodynamically significant bradycardia (13).

In our current approach, atropine test was carried out after 4-hours fast with up to 2 mg intravenous atropine sulfate, under continuous electrocardiogram recording for 30 min, at least 24 hours before the procedure. In patients with AVB, demonstration of 1:1 AV conduction or a decrease more than 25% in PR interval was accepted as a positive response. Post-atropine final sinus rate and PR interval were also used as targeted clinical endpoints after completion of CNA.

### Case 1 presentation (continued)

**Figure 3:**
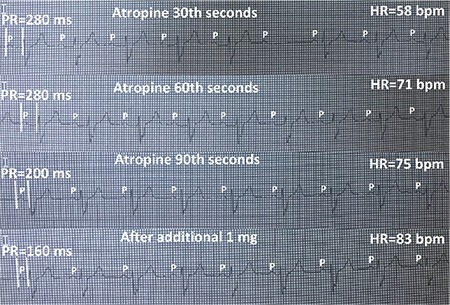
**Successive ECG recordings of case 1 during atropine administration.** After intravenous administration of 0.04 mg/kg atropine, an increase both in sinus rate and AV conduction seen in successive ECGs. AV: atrioventricular; ECG: electrocardiography

Atropine challenge caused a stepwise increase in sinus rate from 58 bpm to 83 bpm, resolution of Mobitz type I AVB, and a stepwise decrease in PR interval from 280 ms to160 ms in successive electrocardiograms (Figure 3).

**Figure 4:**
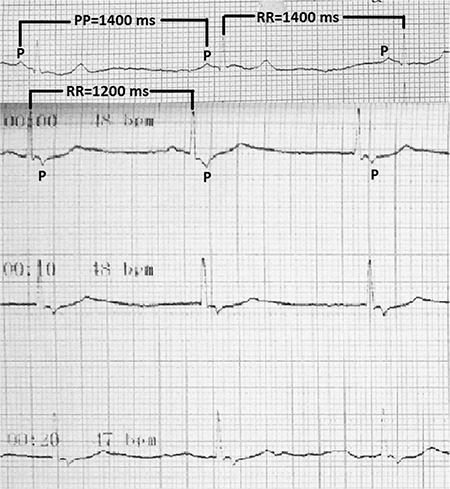
**Successive ECG recordings of a patient demonstrating amiss response to atropine administration.** After intravenous administration of 0.04 mg/kg atropine, junctional rhythm with 1:1 ventriculoatrial conduction is seen. ECG: electrocardiography

Complete resolution of AVB during atropine challenge is a good indicator to exclude functional AVB from intrinsic one, be the AVB paroxysmal or persistent; whereas, unresponsiveness or poor response to atropine should be considered as a potential clue of structural disease of the AV node. Other than that, atropine test may cause amiss response like junctional rhythm in some cases (Figure 4). In existence of structural involvement of sinus node, sinus rate cannot increase after atropine administration. Thus, AV node acts as the dominant pacemaker. Thus, CNA should not be attempted in such cases, as it may cause pacemaker syndrome-like complaints after ablation.

### Case 1 presentation (continued)

Evaluation of the symptom status, Holter findings, and atropine response of the present case revealed it to be a suitable candidate for CNA.

Once a case is decided suitable for CNA, the anatomical and physiological principles of parasympathetic innervation should be considered.

### Anatomical principles of parasympathetic innervation

The intrinsic cardiac nervous system forms a complex neural network composed of GP, concentrated within distinct epicardial fat pads around atria, and contains a heterogeneous population of neurons that include afferent, motor (parasympathetic and sympathetic), and interconnecting local circuit neurons. Theoretically, if we can determine the exact locations of GP, ablation at these sites may permanently damage postganglionic neuronal bodies of the parasympathetic system; whereas, sympathetic and sensory systems will not be permanently affected, because they have only postganglionic nerve fibers in this region and may be repaired by axonal regeneration process over a long-term (14).

**Figure 5:**
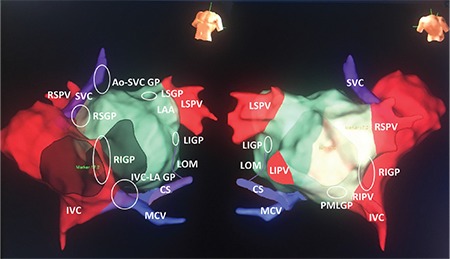
**Electroanatomical illustration of GPs according to our current approach.** Please see the text for details. Ao: aorta; CS: coronary sinus; GP: ganglionated plexi; IVC: inferior vena cava; LAA: left atrial appendage; LIGP: left inferior GP; LIPV: left inferior pulmonary vein; LOM: ligament of Marshall; LSGP: left superior GP; LSPV: left superior pulmonary vein; MCV: middle cardiac vein; RIGP: right inferior GP; RSPV: right superior pulmonary vein; RIPV: right inferior pulmonary vein; SVC: superior vena cava

According to anatomists, there is still no consensus among the authors on the anatomical location and number of GPs. In an animal study, Chiou et al. (15) defined three main parasympathetic GPs located outside the atrial wall in para-cardiac fat pads (Figure 5). Most efferent vagal fibers to the atria travel through aorta–superior vena cava (Ao–SVC) fat pad, located between the medial SVC and the aortic root, superior to the right pulmonary artery; then project onto inferior vena cava–left atrium (IVC–LA) fat pad; the right pulmonary vein (PV) fat pad; and to both atria. According to this classification, Ao–SVC fat pad is the “head station” of vagal fibers traveling to both atria and to the sinus and AV nodes. Bilateral vagal fibers to the sinus and AV nodes also converge first at this ganglion (a few fibers to the sinus node go directly to the right PV fat pad) and then project to the right PV GP and IVC–LA fat pad, which provide vagal innervation to the sinus and AV nodes, respectively. In a human autopsy study, atrial GPs were identified at five distinct localizations (Figure 5): (1) right superior-anterior GP, (2) left superior GP, (3) right inferior-posterior GP, (4) posteromedial left GP, and (5) posterolateral left or left inferior GP (16). Despite existence of such a nomenclature difference between the studies, there is a considerable overlap as shown in Figure 5. Thus, in our current approach, we use following 6 GPs based definition strategy according to ablation order: left inferior GP, left superior GP, right superior GP, right inferior GP, posteromedial left GP, and Ao–SVC GP.

### Physiological principles of parasympathetic innervation

Parasympathetic innervations of sinus and AV nodes demonstrate a selective pattern. Billman et al. (17) studied these in a non-human primate heart. The cervical vagi were electrically stimulated (20 Hz, 4 V, 2 ms) before and after selective denervation of the AV and/or sinoatrial nodal regions. Vagal stimulation was repeated during atrial pacing to assess the parasympathetic modulation of AV nodal conduction. Ablation of parasympathetic pathways to the AV node was accomplished by the disruption of the epicardial fat and surface muscle layer at the junction of the IVC and inferior LA (IVC–LA GP), without affecting the heart rate response. In sharp contrast, surgical dissection of the fat pad overlying the right PV–SVC junction (Ao–SVC and the right superior GP) significantly attenuated negative chronotropic effects of vagal stimulation. These data demonstrate that discrete vagal efferent pathways innervate both nodal regions of a non-human primate heart. Although there is no clear human data, according to experimental studies, the Ao–SVC GP behaves like a head station of all cardiac vagal innervation; whereas, the right superior GP and posteromedial left GP mainly provide innervation to sinus and AV nodes, respectively. Based on this classical anatomical knowledge, the Ao–SVC GP, right superior GP, and posteromedial left GP should be targeted in a patient with AVB, at least.

### Detection of localizations of ganglionated plexis during electrophysiological study

Despite anatomical assumptions, we need a well-defined method to locate the GPs during electrophysiological study, as the localizations may vary significantly between subjects.

**Figure 6:**
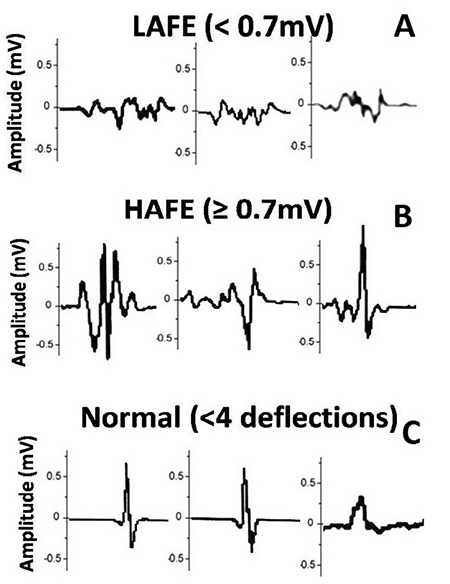
**Different atrial EGM types during sinus rhythm.** Please see the text for details. EGM: electrogram; HAFE: high-amplitude fractionated electrogram; LAFE: low-amplitude fractionated electrogram

We recently defined a fractionated electrogram (EGM)-based ablation strategy and named the technique as electroanatomic-mapping-guided CNA (11). According to this new approach, after creating 3-dimensional electroanatomic maps of both atria using EnSite Mapping System (EnSite Velocity, St Jude Medical, Abbott, Sylmar, CA, USA), bipolar endocardial atrial EGMs are evaluated for amplitude and number of deflections at filter settings of 200-500 Hz and a sweep speed of 400 mm/s. All EGMs are divided into 3 subgroups: (1) normal atrial EGM demonstrating deflections <4; (2) low-amplitude fractionated EGM (LAFE) demonstrating deflections ≥4 and amplitude <0.7 mV; and (3) high-amplitude fractionated EGM (HAFE) demonstrating deflections ≥4 and an amplitude ≥0.7mV (Figure 6). The sites demonstrating HAFE or LAFE pattern in a region that is consistent with probable localization of GPs are tagged as ablation targets.

### Case 1 presentation (continued)

### Details of mapping

A "3-catheter" approach was used for initial electrophysiological study and CNA. With this aim, 3 femoral vein punctures were performed for insertion of a deca-polar 6F steerable electrode catheter into the coronary sinus (CS), a quadripolar electrode catheter into the His region, and an irrigated ablation catheter into the right atrium or LA, depending on the mapping and ablation site. Suprahisian Wenckebach type AVB was confirmed, and sustained arrhythmias, primary conduction system dysfunction, and sick sinus syndrome were ruled out during conventional electrophysiological study. Anatomical shells of the right atrium, the SVC, IVC, and CS were created using Cardiac Mapping System (EnSite Velocity, St Jude Medical, Abbott, Sylmar, CA, USA) before left atrial mapping.

Creation of anatomical shell of right atrial structures before left atrial ablation is important as it demonstrates relationship between right and left atrial structure and is used to define target area in left atrial side (relationship between the right superior PV and the SVC is important during mapping of the right superior GP, and relationship between the CS and the LA is important during mapping of the posteromedial left GP).

### Case 1 presentation (continued)

Transseptal puncture was performed by scopy guidance. Unfractionated heparin was infused (100 IU/kg bolus) intravenously, and the activated clotting time, measured every 30 min, was maintained between 300 and 400 s. After creating a 3-dimensional anatomic map of LA and PVs, fragmented bipolar endocardial atrial EGMs in a region consistent with probable localization of GPs were tagged as ablation targets with previously defined filter settings (Figure 6). Other sites demonstrating LAFE pattern were accepted as scar tissue and were excluded from the assessment.

Although fractionated potentials can be divided as high- or low-amplitude, such a differentiation is not an obligation because all fractionated EGMs are potential targets for ablation. Thus, the actual important point is proper detection of fragmented sites.

There might be two potential concerns related to the usage of fragmentation during GP detection. The first is an increasing amount of fibrosis within the atrial wall that may cause formation of a substrate favorable for slow conduction and display EGMs with lower voltage and fragmentation (18). However, Jadidi et al. (19) demonstrated that most fractionated EGMs are not related to areas of fibrosis and occur in healthy atrial tissue without evidence of fibrosis on magnetic resonance imaging. In the current cases, although magnetic resonance imaging was not applied to exclude structural heart disease responsible for conduction disturbances, this possibility should be kept in mind, especially in older cases. The second concern is that the excessive force of catheter tip on the atrial tissue which does not stretch may lead to misdiagnosis of fractionated EGM, if not using contact-force catheters (20). However, such an excessive force usually causes catheter-related atrial extra-beats. Once catheter stability is achieved, normal atrial EGMs can be seen in the same area.

### Case 1 presentation (continued)

### Details of ablation

**Figure 7:**
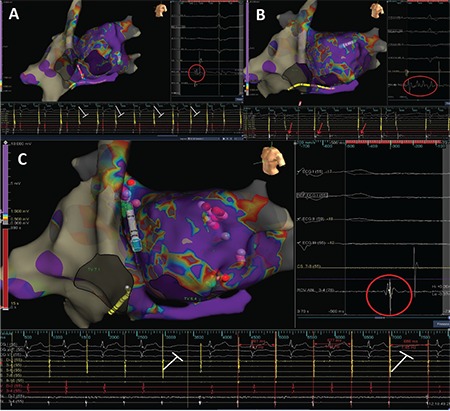
**a-c. Electroanatomical mapping views and intracardiac EGMs during GP ablation.** During the first radiofrequency application on the left inferior GP, Wenckebach block turns to 2:1 AVB due to vagal response (a). Ablation of the left superior GP causes a significant decrease in sinus rate detected during radiofrequency application (b). Unlike other GP sites, ablation of the right superior GP increases sinus rate immediately without affecting AV conduction (c). AV: atrioventricular; EGM: electrogram; GP: ganglionated plexi

Radiofrequency current was applied in a point-by-point fashion in power-controlled mode with an open irrigated-tip catheter (Theraphy Coolflex, St. Jude Medical Inc., St. Paul, MN, USA). Radiofrequency energy was started with 30 watts (W) and limited to 35 W along the roof side, under an irrigation flow rate of 17 mL/min, and to 40W in the remaining areas. Continuous flow during mapping was 2 mL/min. Ablation was started from fragmented EGMs on the left inferior GP site. During the first radiofrequency application at that site, Wenckebach block turned to 2:1 AVB due to vagal discharge (Figure 7a). Ablation was continued until all targeted EGMs were eliminated. The catheter in the His region was left ready for advancement to the right ventricle for back-up pacing in case of significant asystole or high-degree AVB. Once complete elimination was achieved in the targeted EGMs, subsequent radiofrequency applications did not cause new vagal response at the same site.

Continuation of positive vagal response during radiofrequency application should be accepted as a clue for partial denervation, and ablation should be continued in that area until vagal response disappears.

### Case 1 presentation (continued)

We continued the ablation on the left superior GP. Again, all fragmented EGMs were targeted and ablated. Ablation at that site caused a significant vagal response with sinus pause (Figure 7b). The right superior GP was the next station. During radiofrequency application on the right superior GP, level of AVB did not change despite acceleration of the sinus rate (Figure 7c). The ablation catheter was retracted along the interatrial septum and all fragmented areas were ablated on the right inferior GP. Ablation on this GP did not cause any effect on sinus rate and AV conduction.

As discussed before, the right superior GP mainly controls the function of the sinus node, and high frequency stimulation (HFS) of this GP reduces sinus rate without affecting AV nodal conduction (21). A similar observation was recently published by Hu et al. (22) who demonstrated that during ablation of the right superior GP, heart rate increased, while there was just vagal response observed during ablation of other GPs. In the previous animal and human studies, HFS at the left inferior GP or the left superior GP failed to elicit a vagal response if right sided GPs were ablated first (23,24).

### Case 1 presentation (continued)

Our last station in the LA was the posteromedial left GP. Ablation in this GP caused a partial improvement in AV conduction, but 1:1 conduction could not be achieved (Video 1).

Although the right superior GP is suggested as a primary target for CNA, because ablation on this site causes more increase in heart rate than other GP sites, we should focus on the parasympathetic innervation of the AV node in a patient with AVB. In an animal study, Randall et al. (25) demonstrated that GPs supplying the AV are found within a smaller fat pad overlying epicardium at the junction of the IVC and the LA. Also, Armour et al. (16) demonstrated that the largest number of ganglia, an average of 194 of the 458 ganglia per heart, is located on the posterior surface of the right atrium adjacent to the interatrial groove, and this ganglion contains more neurons compared with other ganglia. Anatomically, those sites correspond to ganglion C, the posteromedial left GP, and slightly to the right inferior GP (Figure 5).

### Case 1 presentation (continued)

In the following steps of the procedure, we targeted right atrial part of GPs according to our ablation order. In the right atrium, Ao–SVC fat pad and right part of the right superior GP was targeted and ablated.

The distance between the left and the right endocardium surrounding the right superior GP is 3±1 mm. Therefore, bi-atrial ablation may increase the possibility of creation of a transmural ablation. According to our experience, similar fractionated EGMs can be detected on lower part of SVC; whereas, on upper side, it is not always possible to detect EGMs. Therefore, we suggest performing empirical ablation between the aortic root and the medial SVC when the targeted sinus rate is not achieved.

### Case 1 presentation (continued)

**Figure 8:**
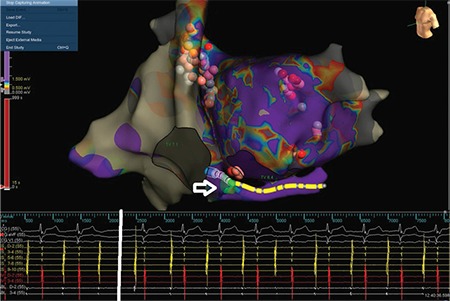
**Electroanatomical mapping views and intracardiac EGMs seen during ablation on the inferior vena cava-left atrium GP.** During the first radiofrequency application on the posteromedial left GP, a 1:1 AV block is gained. Please see Figure 5 for comparison. AV: atrioventricular; EGM: electrogram; GP: ganglionated plexi

**Figure 9:**
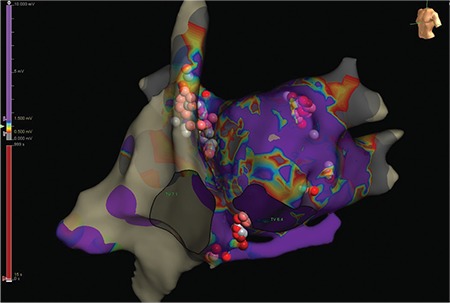
**Final ablation lesion sets shown on electroanatomical mapping system. Red, pink, and white spheres demonstrate ablation lesions.** Fragmented EGMs recorded at distal tip of ablation catheter are indicated with red circles. EGM: electrogram

**Figure 10:**
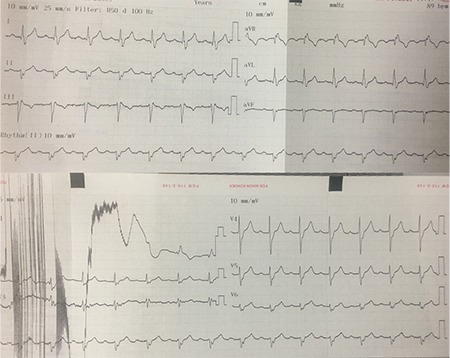
**Post procedural resting ECG of case 1. A 1:1 AV conduction with sinus rate of 89 bpm is seen.** Please compare post-procedure ECG with ECG after atropine administration. AV: atrioventricular; ECG: electrocardiography

In that step of the procedure, AVB was still present. So, we decided to target the IVC–LA GP site located around the CS ostium (Figure 5). A 1:1 AV conduction was achieved after a couple of ablation lesions in the CS ostium (Figure 8, Video 2). Figure 9 and 10 demonstrate final lesion sets and post-procedure rest electrocardiogram with 1:1 AV conduction, respectively.

However, it should be kept in mind that discrimination of fragmented EGMs related to vagal innervation from other fragmentation causes such as slow pathway or double potentials is not easy during right sided ablation around CS (26). Thus, we suggest starting ablation from the posteromedial left GP, firstly. If a 1:1 AV conduction can be achieved after ablation of other GP sites, ablation should be avoided around the CS. However, in the present case, ablation was performed around the CS because the desired target could not be met.

## CASE 2 PRESENTATION

**Figure 11:**
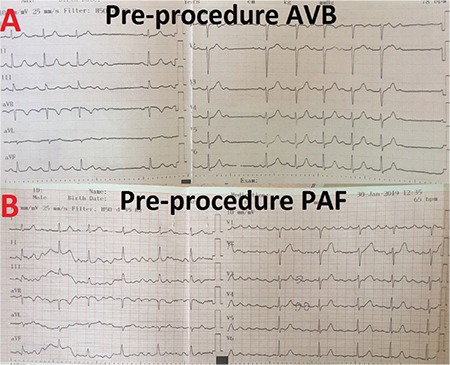
**a, b. Pre-procedure ECGs of case 2 during the complaint.** Mobitz type I AVB is seen during resting ECG (a). AF with low ventricular rate due to vagal activation is seen (b). AVB: atrioventricular block; ECG: electrocardiography

A 56-year-old female was admitted to our clinic with frequent palpitations and episodes of presyncope. Mobitz type I AVB and atrial fibrillation (AF) with low ventricular rate were detected on successive rest electrocardiograms (Figure 11).

Again, in the first step, it is important to determine nature of AVB and AF episodes (i.e., vagally mediated or not). With this aim, the electrocardiogram characteristics before, during, and after AF episodes should be carefully examined on continuous Holter tracings.

### Case 2 presentation (continued)

**Figure 12:**
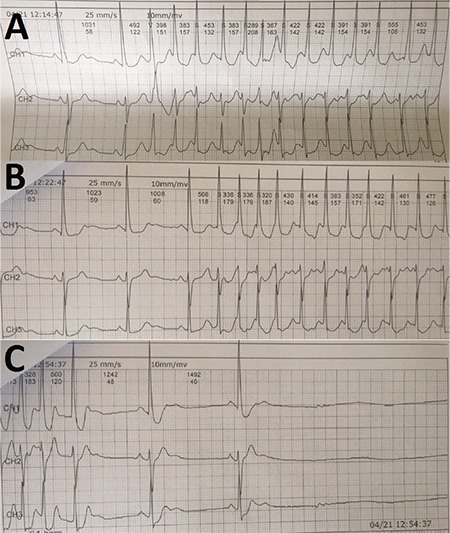
**a-c.** Holter ECG tracings of case 2 show AF and sinus pause episodes. For details, please see the text. AF: atrial fibrillation; ECG: electrocardiography

AF was preceded by sinus node slowing and ended with sinus bradycardia and asystole (Figure 12). Acceleration of sinus rate and AV conduction was confirmed by atropine test. Therefore, we decided to perform CNA in addition to pulmonary vein isolation in this case.

### Details of ablation

**Figure 13:**
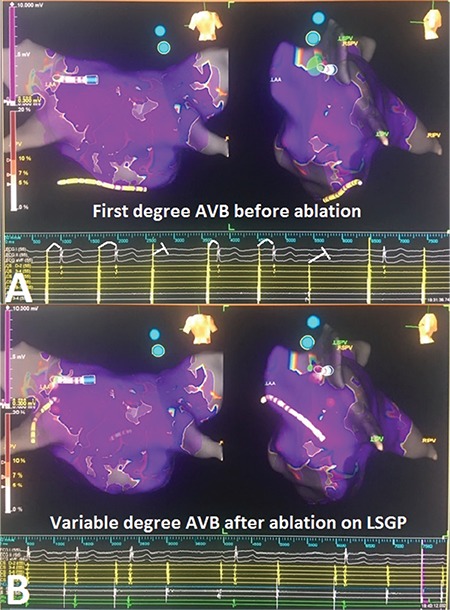
**a, b. The 3-dimensional electro-anatomical mapping views of both atria and the CS.** Before radiofrequency ablation on the LSGP, Mobitz type I AVB with stepwise increase on PR interval is seen in intracardiac EGMs (a). During radiofrequency application on LSGP, variable degree AVB is seen due to vagal discharge (b). AVB: atrioventricular block; EGM: electrogram; GP: ganglionated plexi; LSGP: left superior GP

**Figure 14:**
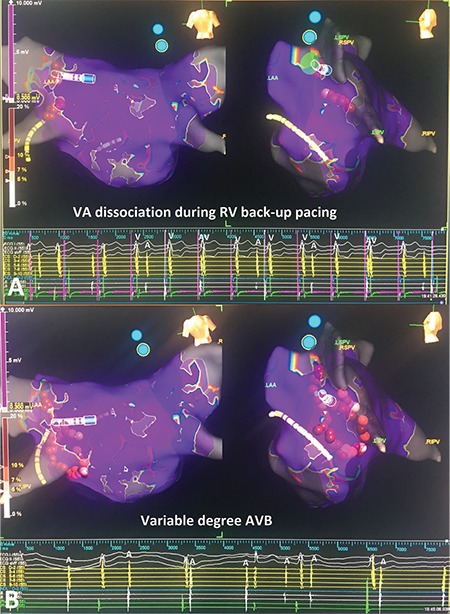
**a, b. Successive 3-dimensional electro-anatomical mapping views of case 2.** During RV back-up pacing VA dissociation is seen due to complete VA block (a). Pink lines demonstrate pacing spikes. Please see that AA intervals are constant due to VA block during pacing. After cessation of pacing, maintenance of variable degree AVB is seen (b). AVB: atrioventricular block; RV: right ventricular; VA: ventriculo-atrial

**Figure 15:**
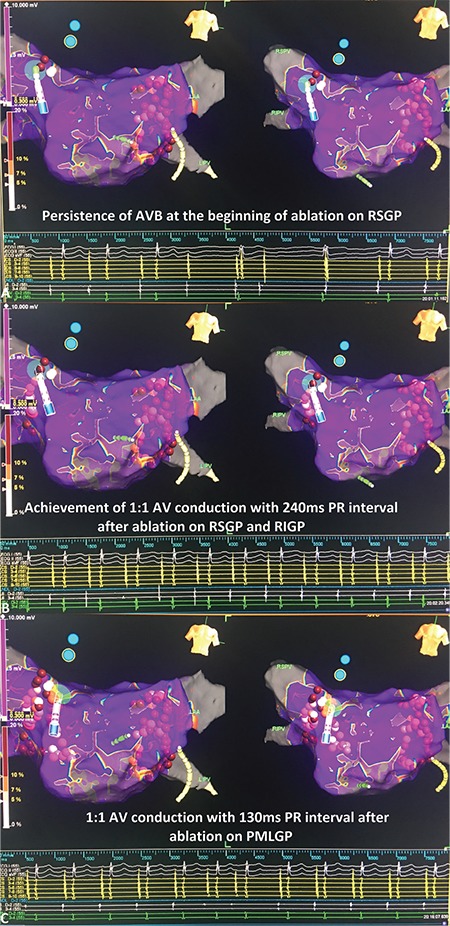
**a-c. Following 3-dimensional electroanatomical mapping views of case 2.** At the beginning of ablation on the RSGP, persistence of AVB is seen in intracardiac EGMs (a). After completion of ablation on RSGP and on the RIGP, achievement of 1:1 AV conduction is seen in intracardiac EGMs. However, PR interval is 240 ms which is compatible with the first degree AVB (b). Ablation of the posteromedial left GP causes a significant shortening of PR interval (c). AV: atrioventricular; AVB: atrioventricular block; EGM: electrogram; GP: ganglionated plexi; RSGP: right superior GP; RIGP: right inferior GP

After creating a 3-dimensional electroanatomic mapping of both atria, fragmented EGMs on the left superior and left inferior GPs were ablated, respectively. During the first radiofrequency application in the left superior GP, Wenckebach block turned to high-degree AVB due to vagal discharge (Figure 13, 14). The procedure was continued with circumferential ablation lesions at least 1 cm outside the left PV ostia to encircle and electrically isolate the ipsilateral pairs. Then, the superior right, inferior right, and posteromedial left GPs were evaluated for HAFEs and LAFEs and ablated (Figure 15). The procedure was continued with circumferential lesions at least 1 cm outside the right PV ostia to encircle and electrically isolate the ipsilateral pairs. Radiofrequency energy was delivered with power of up to 35 W, a maximum flow rate of 30 ml/min, and a maximum temperature of 43°C. Radiofrequency power was limited to 20-25 W on the posterior wall. A 1:1 AV conduction was achieved after ablation on the posteromedial left GP (Figure 15). Therefore, we ended the procedure without performing additional ablation via right atrial side.

## DISCUSSION

Until now, no consensus has been achieved about an optimal technique to define localization of vagal innervation sites. Different groups have defined different approaches based on positive response with HFS, EGM characteristics, or anatomical landmarks (27,28,29,30,31,32). Theoretically, application of HFS causes 2 different responses in the atria: (1) a vagal response diagnosed with a significant prolongation of the PR or RR intervals; (2) a normal response characterized by the absence of any effect or non-significant changes on the PR or RR intervals. These responses demonstrate vagal innervation sites and normal atrial myocardium, respectively. The HFS-based CNA was firstly studied by Yao et al. (27). Although different protocols have been used in studies, the stimulation is usually delivered with a frequency of 20 Hz, amplitude of 0.1-1 mA or 10-30 V, and pulse duration of 1-10 ms for 5 seconds at each site. However, HFS may cause sensations of discomfort, especially with increasing voltage, due to a specific excitation of nociceptors within the wall of the atria, SVC, or CS. Therefore, conscious patients cannot tolerate stimulation of more than 15 V. Also, applications with high amplitude may cause inadvertent AF induction. In a recently published study, Sun et al. (28) compared HFS-guided strategy with empirical anatomic ablation and found similar success rates. Low specificity of HFS response may be one of the potential explanations to this result.

By using spectral analysis, Pachon et al. (29) defined 2 different EGM types: (1) fibrillar atrial myocardium, which demonstrates vagal innervation sites and exhibits a typically highly fragmented, heterogeneous, and right-skewed frequency distribution; and (2) compact atrial myocardium, which is related to normal atrial myocardium and presents homogeneous and fast conduction with left-skewed frequency distribution. The only disadvantage of this technique seems to be the need to scrutinize the whole atrial endocardium using computer-aided mapping. There is still no software suitable with currently available 3-dimensional mapping systems.

Anatomical ablation as a stand-alone strategy has been used by different groups (30,31,32). Although there is no clear data for AVB cases, in a recently published meta-analysis, we compared recurrence rates between different approaches in patients with vasovagal syncope. From the survival plot, anatomically based CNA was associated with higher rates of syncopal recurrence in patients with vasovagal syncope (7).

To deal with potential limitations of those 3 techniques, we defined a new and relatively simple strategy. Twelve of the patients with vasovagal syncope underwent CNA with this recently introduced electroanatomic-mapping-guided strategy, while 8 patients underwent combined CNA using a combination of HFS and spectral analysis (6). Compared with the combined approach, electroanatomic-mapping-guided CNA exhibited a shorter procedure and fluoroscopy times. Median event-free survival was comparable between the groups. Efficiency of the technique was also confirmed in a patient with functional AVB (11).

Additionally, there is no consensus on how to perform the procedure, as whether ablating all right and left atrial GP areas; or a selective ablation in innervations sites of the sinus and AV nodes, in both sides of the septum; or only the right side of the septum; or even spots in the SVC (4,5,6,27,28,29,30,31,32). Present two cases demonstrate that ablation approach may be changed from one patient to another according to the indication and the desired endpoint. Despite that fact, CNA still has no class of recommendation in guidelines. The main reason for this is lack of large randomized studies evaluating long follow-up and procedural risk. There are some trials underway (NCT03903744, ABSTRACT Registry) which will shed more light on CNA; however, none of them prospectively compare different techniques used during this procedure.

In a recently published article, the most common place of vagal response during ablation was the left superior GP (22). A similar trend was also seen in current cases. However, in some patients, the sinus node artery may be located in this area (33). Thus, ablation in this place may provoke coronary artery spasm and cause asystole, which may be misinterpreted as a vagal response. However, bradycardia related to injury of the sinus node artery usually occurs more than 24 hours after ablation.

Furthermore, there is still no well-defined procedural endpoint. Reaching an expected heart rate elevation such as that seen during pre-procedure atropine administration, elimination of all positive vagal response induced by HFS on the target sites, or an absence of an additional heart rate elevation after ablation with the atropine test has been used with this aim. In case of AVB, achievement of 1:1 AV conduction should be the first clinical endpoint. Also, absence of an additional shortening of atrium–His interval after ablation with the atropine test may be used to demonstrate complete vagal denervation. Although a negative response to atropine clearly demonstrates denervation, it is necessary to wait at least 4 hours for the effect of atropine to resolve if the positive response continues. Therefore, we need a more objective, faster, and reproducible technique to evaluate the ablation effect during the procedure. Recently, Pachon et al. (34) defined a technique based on extracardiac vagal stimulation through internal jugular vein that allows repeated evaluation of vagal denervation at any step of ablations without causing persistent autonomic modification.

Based on all the evidence, ablation of GP may markedly improve health and quality of life in patients with pronounced symptoms and in those who cannot be treated effectively with drugs or refuse permanent pacemaker implantation. There was no reported major complication related to procedure. However, the procedure carries some risk of serious complications as septal puncture. In our cohort consisting of 20 vasovagal syncope cases, inappropriate sinus tachycardia was seen in 2 cases (6). The arrhythmia was symptomatic in one of these patients. Symptoms were successfully eliminated with ivabradine. Nevertheless, the proarrhythmic effect of the GP ablation still needs to be addressed in further studies. Hence, it is important that patients who are candidates for treatment receive comprehensive and objective information concerning the risks and the expected benefits of the method.

CNA might be a feasible and valuable adjunctive therapy in well-selected functional AVB cases. Instead of complex spectral analysis or HFS application, analysis of EGM characteristics may be used to define vagal innervation sites during electrophysiological study. Currently, there are still more questions than answers concerning the efficacy and safety of the method. Results of randomized studies will determine indications for CNA in patients with vasovagal syncope and conduction disturbances owing to enhanced parasympathetic activity.

**Video 1.** DOI: 10.4274/balkanmedj.galenos.2019.2019.9.047.video1

**Video 2.** DOI: 10.4274/balkanmedj.galenos.2019.2019.9.047.video2
